# Soil‐transmitted helminth parasites and allergy: Observations from Ecuador

**DOI:** 10.1111/pim.12590

**Published:** 2018-10-17

**Authors:** Martha E. Chico, Maritza G. Vaca, Alejandro Rodriguez, Philip J. Cooper

**Affiliations:** ^1^ Fundación Ecuatoriana Para Investigación en Salud Quito Ecuador; ^2^ Faculty of Epidemiology and Population Health London School of Hygiene and Tropical Medicine London UK; ^3^ Facultad de Ciencias Medicas, de la Salud y la Vida Universidad Internacional del Ecuador Quito Ecuador; ^4^ Institute of Infection and Immunity St George's University of London London UK

## Abstract

There is considerable interest as to potential protective effects of soil‐transmitted helminths (STH) against allergy and allergic diseases. Here, we discuss findings of studies done of the effects of STH parasites on atopy and allergic diseases in Ecuador. While cross‐sectional studies have consistently shown a reduced prevalence of allergen skin prick test (SPT) reactivity among infected schoolchildren, the removal of these infections by repeated deworming did not affect SPT prevalence over the short‐term (ie, 12 months) but may have increased SPT prevalence over the long‐term (ie, 15‐17 years). In the case of allergic symptoms, cross‐sectional studies have generally not shown associations with STH and intervention studies showed no impact on prevalence. However, a birth cohort suggested that early STH infections might reduce wheeze by 5 years. Allergic sensitization to *Ascaris*, however, explained a significant proportion of wheezing among rural schoolchildren. Studies of the effects of STH on immune and inflammatory responses indicated a potential role of STH in contributing to more robust regulation. The effects of STH on allergy are likely to be determined by history of exposure over the life‐course and by interactions with a wide variety of other infectious and non‐infectious factors.

## 1 INTRODUCTION

Over the past 20 years we have conducted a programme of research in Ecuador exploring how helminths may affect the development of allergy among children. Our research followed observations made in Africa^1^ and a highly influential series of studies published during the 1990s by Lynch and colleagues in Venezuela.^2^, ^3^, ^4^, ^5^ The Venezuelan studies and others that followed elsewhere^6^, ^7^, ^8^ showed variable but measurable effects of soil‐transmitted helminth (STH) or geohelminth infections on allergen skin test reactivity and asthma symptoms, and were done in the context of a growing interest in the hygiene hypothesis and potential protective effects against allergy of childhood infections.^9^

**Figure 1:**
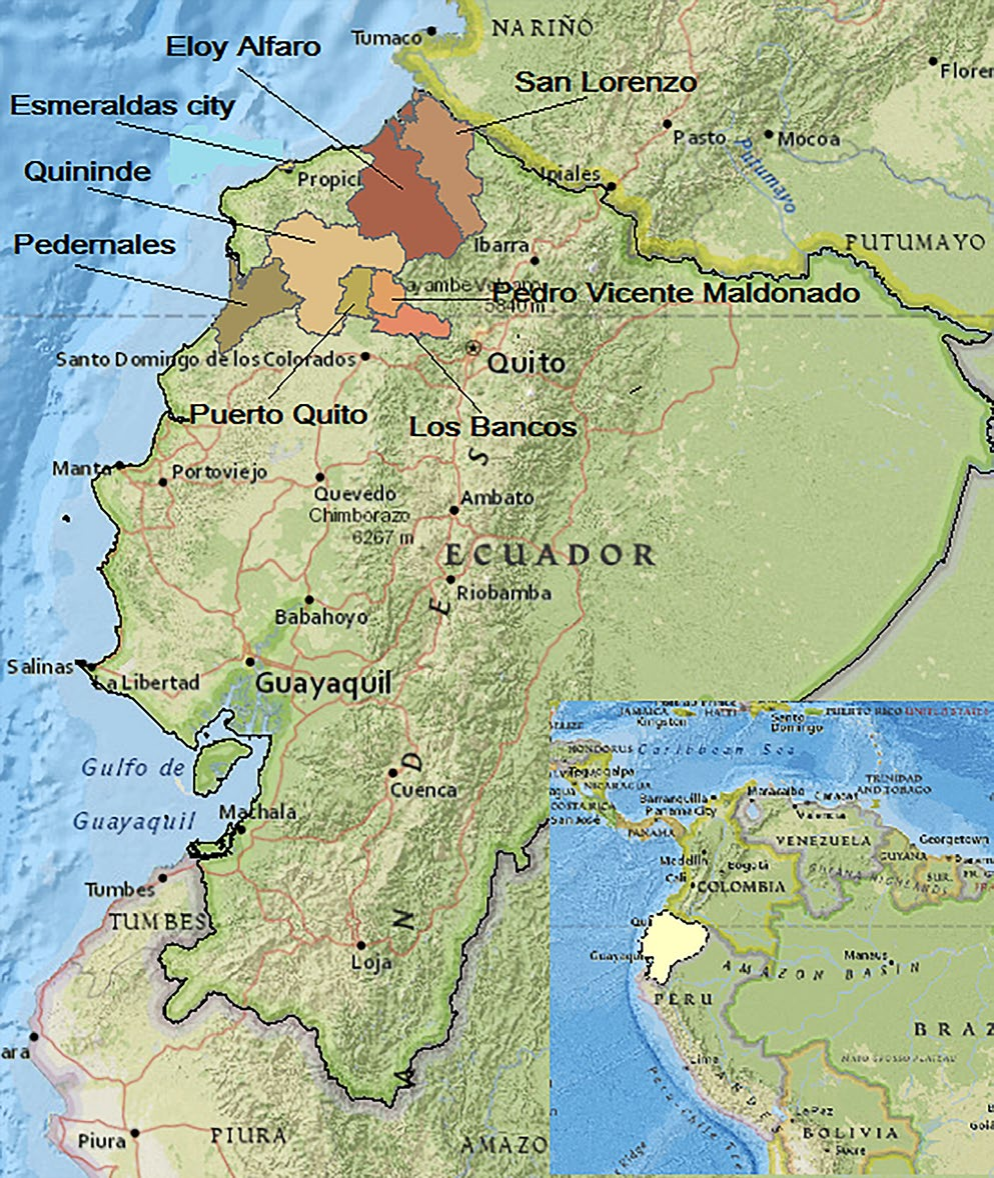
Map of Ecuador showing locations where studies of soil‐transmitted helminth infections and allergy were done. Studies were done in tropical and sub‐tropical regions of: Esmeraldas Province in the City of Esmeraldas,^30^, ^53^, ^54^, ^77^, ^78^, ^81^ the Districts of Quininde,^26^, ^38^, ^40^, ^81^ and Eloy Alfaro^27^, ^30^, ^34^, ^41^, ^53^, ^55^, ^77^, ^78^, ^81^ and San Lorenzo^30^, ^34^, ^41^, ^53^, ^55^, ^68^, ^74^, ^77^, ^78^; Pichincha Province in the Districts of Pedro Vicente Maldonado, Puerto Quito and San Miguel de los Bancos^26^, ^27^, ^28^, ^29^, ^61^, ^62^, ^66^; and Manabi Province in the District of Pedernales^60^

In this review, we present an overview of the findings of epidemiological and immunological studies done in Ecuador to understand better the effects of STH parasites on allergy during childhood. We will present current evidence for effects of STH parasites on atopy and allergic symptoms, focussing largely on the findings of studies done in Ecuador, and referring where relevant to the wide body of research that has been done elsewhere in Latin America and other endemic regions for STH parasites. The review will show that evidence for a protective effect of STH parasites against allergy in children remains fragmentary and inconsistent and that, while STH parasites do modulate the host anti‐parasite immune response, there is limited evidence that they play a critical role in modulating the host inflammatory responses that may lead to allergic diseases in humans (Figure 1).

## 2 SOIL‐TRANSMITTED HELMINTHS

Infections with the common STH parasites (*Ascaris lumbricoides, Trichuris trichiura* and hookworm) are estimated to infect 1.5 billions worldwide,^10^ primarily in poor tropical and sub‐tropical regions among populations living in poverty and inadequate sanitation. STH infections have been associated with significant disability and mortality, particularly in children.^10^ The World Health Assembly (WHA) in 2001 endorsed a strategy for the control of these parasites through periodic treatments of schoolchildren with anthelmintic drugs.^11^ In line with this resolution, WHO, national governments and donor organisations have prioritised the control of STH infections through periodic treatments with anthelmintic drugs to school‐age children. Subsequently, recommendations for preventive chemotherapy have been expanded to include other high‐risk groups, namely pre‐school children and women of childbearing age.^12^, ^13^

The prevalence of STH infections is likely to have declined significantly in many low and middle‐income countries (LMICs) over the past 10‐20 years through reductions in extreme poverty, access to improved sanitation and increasing coverage rates for preventive chemotherapy for high‐risk groups. Nevertheless, regions of high STH prevalence are likely to persist among populations that continue to suffer high levels of poverty and which are geographically isolated. A recent national survey of STH prevalence in Ecuador that studied a representative sample of schoolchildren estimated a national prevalence of 28%,^14^ considerably lower than previous estimates, reflecting substantial progress over the previous 10 years in achieving the millennium development goals. However, the Amazon region of Ecuador continued to have high prevalence rates (59%) reflecting the relative isolation of this population that continues to suffer high levels of poverty^14^, ^15^

## 3 ALLERGY

Asthma and eczema, considered to be caused by inflammatory mechanisms, are among the commonest of all chronic diseases of childhood in industrialized countries. Asthma alone is estimated to affect more than 300 millions worldwide^16^ and has emerged as an important public health problem in many non‐industrialized regions, particularly in growing cities and among urbanizing populations.^16^, ^17^, ^18^

Temporal trends of increasing allergic disease prevalence are thought to be explained by a changing living environment, perhaps related to a decline in the incidence of childhood infectious diseases and reduction in the diversity of environmental microbiota.^19^ Rural residence has consistently been shown to be protective against allergy^18^, ^20^ but it is not clear which rural exposures may mediate this protection. Numerous studies in traditional farming settings in Europe have shown farming to be strongly protective against atopy and allergic diseases,^21^ particularly if farming exposures occur in early life. In such settings, protection appears to be partly dependent on exposures to a diverse range of microorganisms in the environment.^22^ Protection against allergy is thought to be strongest when protective exposures occur during early life^23^, ^24^ while the immune system is still developing and may allow the developing immune response to develop a greater capacity to regulate inflammation. Such exposures are likely to be multiple, are likely to vary according the environment in which a child is raised and may include helminths.

## 4 STH PARASITES AND ALLERGY

The apparently low prevalence of allergy in the rural tropics may be explained partly by endemic helminth infections. In order to survive for periods of years in their mammalian hosts, helminth parasites have developed immune mechanisms that modulate the inflammatory responses that the host has evolved to kill them.^25^ Such inflammatory responses include key elements of Th2‐mediated allergic inflammation (eg, IgE, mast cells, eosinophils, Th2 cells, etc). Helminths may suppress not only these killing and expulsion mechanisms but also shared inflammatory pathways that lead to atopy and allergic diseases. A protective role for STH and other helminth parasites against allergy has been incorporated into an extended hygiene hypothesis that attributes protection against allergy not only to early childhood infections (eg, bacteria and viruses) but also parasites and a more diverse host microbiota.^26^ The specific effects of STH parasites may depend on age of first exposure, intensity of infection and species of infecting parasite. We will now consider the findings of epidemiological studies done in Ecuador examining the effects of STH parasites on atopy and allergic diseases.

## 5 EFFECTS OF STH PARASITES ON ALLERGY IN ECUADOR

### 5.1 STH and atopy

We have repeatedly observed, in a series of cross‐sectional studies in tropical and sub‐tropical regions of the Ecuadorian Provinces of Esmeraldas and Pichincha, an inverse association between the microscopic presence of STH parasites in stool samples and atopy measured using skin test reactivity to environmental allergens.^26^, ^27^, ^28^, ^29^, ^30^ These studies were done in schoolchildren with high prevalence rates for STH (~70%), where the dominant parasites were *A. lumbricoides* and *T. trichiura*, and where the background prevalence of SPT was variable (~10% in Esmeraldas^30^ vs ~20% in Pichincha^26^, ^27^, ^28^, ^29^). The findings are summarized in Table 1. These studies showed inverse associations between STH infections and SPT with odds ratios of ~0.7 and independent effects of both *A. lumbricoides* and or *T. trichiura* on SPT with evidence for greater inverse effects at higher parasite burdens for both parasites (Table 1). Further, the presence of anti‐*Ascaris* IgG4 antibodies, used as a marker for chronic STH infection, was inversely associated with SPT.^26^

**Table 1:** Summary of findings of epidemiological studies done in Ecuador of associations between soil‐transmitted helminth (STH) parasites and allergen skin prick test reactivity

Study	Design/population	Sample	Geohelminths	Outcomes	Findings
Cooper et al, 2016^26^	Cross‐sectional, Pichincha	Schoolchildren, 5‐19 y, n = 2865	Active STH; anti‐Ascaris IgG4; total IgE Prevalence: any 69%; Al 55%; TT 46%; Hk 3%	SPT Any 22%; Dp 9%/Df 6%, cock 11%	Inverse associations with: active (any STH OR 0.64, Al 0.74 and Hk 0.64) and chronic (high total IgE; and presence of anti‐*Ascaris* IgG4) infections. Dose‐response for Al and Tt
Cooper et al, 2003^27^	Cross‐sectional, Pichincha & Esmeraldas	Schoolchildren, 5‐18 y, n = 4433	STH prev: any 63%; Al 50%; TT 44%; Hk 2%	SPT Any 18%; hdm 9%, cock 9%)	Inverse association with active STH (any OR 0.62, Al 0.65 and Tt 0.69. Dose‐response for AL and Tt
Cooper et al, 2004^28^	Cross‐sectional, Pichincha	Schoolchildren, 7‐17 y, n = 1002	STH prev: any 70%; Al 52%; TT 52%; Hk 8%	SPT Any 20%; Dp 9%/Df 6%; cock 12%)	Inverse association with SPT (any OR 0.65; Al OR 0.65; Tt, OR 0.67). Dose‐response for Al and Tt. STH effect independent of household crowding and SES
Cooper et al, 2006^29^	Cluster‐randomized trial with bi‐monthly albendazole for 12 mo, Pichincha	Schoolchildren, 7‐17 y, n = 2373 in 68 schools	STH prev: any 72%; Al 56%; Tt 56%; Hk 15%	SPT Any 25%, hdm 10%, cock 17%	Inverse association at baseline (any 0.78). No effect of treatment on atopy
Endara et al, 2010^34^	Cross‐sectional, Comparison of communities receiving ivermectin MDA for 15‐17 y vs non‐MDA communities Esmeraldas	Rural schoolchildren, 6‐16 y, n = 3901	STH prev: any 63 vs 86%, Al 49 vs 57%, Tt 31 vs 82%, Hk 15 vs 4%	SPT Any 17 vs 9%, hdm 8 vs 5%, cock 4 vs 3%	Inverse association with active STH (any OR 0.71, Tt OR 0.72). Dose‐response for Tt. Long‐term MDA associated with higher prevalence of SPT (OR 2.1)
Moncayo et al, 2013^55^	Case‐control, Esmeraldas	Rural schoolchildren, 7‐19 y, n = 376	STH prev: any 72%; Al 47%; Tt 58%; Hk 8%. Anti‐Ascaris IgE 65%	Wheeze cases vs non‐wheeze controls. Atopy/SPT: any 15%, hdm 10%, cock 7% Atopy/sIgE: any 28%, hdm 19%, cock 15%	Presence of anti‐Ascaris IgE (*P* inter = 0.02) but not active STH attenuated association between sIgE and SPT
Cooper et al, 2014^30^	Cross‐sectional, Esmeraldas	Rural and urban schoolchildren, 5‐16 y, n = 6821	Rural prev: any 69%, Al 42%, TT 54%, Hk 6%. Urban: any 43%, Al 20%, Tt 35%, Hk 5%	SPT Rural: any 13%, hdm 7%, cock 5% Urban: any 10%, Hdm 8%, cock 2%	Inverse association with SPT (Al OR 0.73; Tt, OR 0.71). No interactions by urban vs rural
Cooper et al, 2016^26^	Prospective, Esmeraldas	Rural birth cohort to 3 y, n = 2069 (of 2404 recruited)	Maternal prev: any 46%, Al 28%, Tt 29%, Hk 6%	SPT^a^ in children at 3 y: any 17% hdm 9%, cock 3%	No overall effect of maternal STH on child SPT (any allergen) at 3 y. Inverse association with hdm (OR 0.61). Maternal ascariasis associated with reduced SPT (any OR 0.70, hdm 0.48)
Cooper et al, 2018^40^	Prospective, Esmeraldas	Rural birth cohort to 5 y, n = 2090 (of 2404 recruited)	Maternal prev: any 46%, Al 27%, Tt 28%, Hk 6% Childhood prev. to 3 y: any 34%, Al 26%, Tt 17%, Hk 1%	SPT^b^ in children at 8 y: any 14% hdm 8%, cock 4%	Overall, neither maternal nor childhood STH associated with SPT. Childhood STH inversely associated with SPT to perennial allergens (OR 0.70)

SPT, allergen skin prick test reactivity; sIgE, presence of any allergen‐specific IgE (≥0.7 kU/L); anti‐*Ascaris* IgE, presence of specific IgE to *Ascaris* (≥0.7 kU/L); y, years; MDA, mass drug administration or given at community level; Al, *A. lumbricoides*; Tt, *T. trichiura*, Hk, hookworm; prev., prevalence; Dp, *Dermatophagoides pteronyssinus*; Df, *Dermatophagoides farinae*; hdm, house dust mite; cock, cockroach (*Periplaneta americana*); OR, Odds Ratio; *P* inter., *P* value for interaction.

a SPT positivity defined by wheal size ≥2 mm above negative control.

b SPT positivity defined by wheal size ≥3 mm above negative control.

Based on these findings, we hypothesized that STH were actively suppressing SPT and that this suppression might be reversed by curative chemotherapy. To test this hypothesis, we did a cluster‐randomized study in which schoolchildren living in endemic communities were randomized to receive albendazole given every 2 months for a year vs no treatment.^29^ A placebo was not used because of difficulties in blinding mothers to treatment allocation. The study showed no effect on atopy after 12 months of periodic anthelmintic treatment.^29^ The study had several limitations: (a) a period of 12 months may be insufficient to allow reversal of the immunologic mechanism by which STH parasites suppress SPT; (b) single doses of 400 mg of albendazole have limited efficacy against *T. trichiura* infection; and (c) effects on SPT may be parasite species‐specific (eg, the prevalence of hookworm was relatively low [15%] in our study^29^). Randomized intervention studies conducted elsewhere among schoolchildren have shown inconsistent effects on atopy^31^, ^32^, ^33^; a cluster‐randomized study in Indonesia was unable to demonstrate an effect on SPT following periodic anthelmintic treatments given over 21 months^33^ while randomized studies in Vietnam (12 months follow‐up^32^) and Gabon (30 months follow‐up^31^) provided evidence for an increase in the prevalence^32^ or incidence of SPT.^31^ The findings of these studies do suggest that 12 months of periodic chemotherapy is probably sufficient to show an effect^32^ if one truly exists and that hookworm prevalence may not be a critical factor^32^, ^33^—hookworm prevalence in the Indonesian study was >60%.

To look at long‐term effects of anthelmintic treatments on SPT, we analysed a population in Esmeraldas Province where annual or twice‐annual treatments with the broad‐spectrum anthelmintic drug ivermectin had been distributed at a community level for the control of onchocerciasis over the previous 15‐17 years.^34^, ^35^ SPT and STH prevalence was compared between communities that had received ivermectin compared with communities that had not received any mass drug administrations (MDA). We observed a greater prevalence of SPT in treated communities and this effect was partially explained by a lower prevalence of *T. trichiura* infection but not *A. lumbricoides*.^34^ Periodic anthelmintic treatments given over 15 or more years may cut transmission levels and reduce community prevalence of STH and thus attenuate the induction of immune regulatory mechanisms associated with chronic infections. It is possible that *T. trichiura* may be particularly important in mediating an SPT‐suppressive effect in some populations. The higher incidence of SPT in Gabon following anthelmintic treatment was explained partly by effects on *T. trichiura* prevalence,^31^ and a prospective study in Brazil indicated that suppressive effects against SPT in childhood were related to having higher parasite burdens with *T. trichiura* earlier in childhood.^36^

An explanation for our observations that periodic anthelmintic treatments of schoolchildren over 12 months did not affect SPT prevalence^29^ but that periodic mass treatments of communities over 15 years did seem to have an effect,^34^ might be related to the potential importance of early‐life exposures to STH in mediating a protective effect—through either maternal infections and/or early childhood infections. Treatment of schoolchildren may be too late to reverse a programmed effect while the effect of long‐term MDA on STH transmission within communities would be to reduce exposures in mothers and young children^34^—ivermectin MDA was started in endemic communities before the schoolchildren were born.

Environmental exposures that have been associated with protection against allergy such as farming have stronger effects when exposure occurs in utero and in early childhood.^23^, ^24^ In the case of STH infections, early exposures could occur through trans‐placental passage of parasite antigens from the mother to foetus in utero, through breast milk in the infant or through early childhood exposures to STH parasites in the home. Such early exposures could cause immunological sensitization or tolerization to parasite antigens leading to down‐regulation of host anti‐parasite responses and reduced allergy through immunological cross‐reactivity between parasite and environmental allergens or bystander suppression.^37^ Certainly, antigen‐specific T cell responses to *Ascaris* antigens are measurable in newborns of infected mothers indicating in utero sensitization.^38^ To explore the potential role of early‐life exposures to STH parasites on the development of allergy, we followed a birth cohort, the ECUAVIDA cohort, in a rural District in Esmeraldas Province. We measured the presence of STH infections in mothers and their children during the first 5 years of life and monitored the effects of these early exposures on the development of SPT.^39^ Analyses of the cohort to date have shown no significant effects on SPT overall. However sub‐group analyses showed a reduced prevalence of SPT to mite or perennial allergens among 3‐year‐old children who had infected mothers^26^ and among 5‐year‐old children who had STH infections during the first 3 years of life.^40^ Differential effects of parasite species or by parasite burden were not seen. Ongoing analyses of the cohort at 8 years of age are trying to discern the significance of these sub‐group findings over the longer term.

If we are to ignore the findings of sub‐group analyses within the birth cohort which require replication, it would seem overall that, in Ecuadorian populations where we have repeatedly observed inverse associations between STH and SPT, that early‐life exposures to STH or short‐term anthelmintic treatments at school age do not affect SPT prevalence while longer term treatments might, although the latter observation comes with the caveat of potential biases in the study design.^34^ Thus, the strength of evidence for an effect of STH on SPT prevalence in the series of studies done in Ecuador remains limited. Possible alternative explanations are as follows: (a) lack of a true causal association—other environmental factors with which STH infections are associated (ie, broadly associated with poverty) mediate the protective effects; (b) reverse causality—rather than STH protecting against SPT, it is an allergic predisposition measured by SPT that is associated with enhanced host resistance to these parasites.^6^

## 6 STH AND ALLERGIC SYMPTOMS

We examined associations between STH parasites and allergic symptoms (wheezing, eczema and rhinitis) in a series of cross‐sectional studies in Ecuador.^27^, ^30^, ^41^ The findings are summarized in Table 2. Overall, we observed no associations between STH parasites and allergic symptoms and no effects of individual parasite species or by parasite burden.^27^, ^30^ However, in an analysis of schoolchildren in rural Esmeraldas Province we did observe an inverse association between higher parasite burdens with *T. trichiura* infection and atopic wheeze (defined by recent wheeze in the presence of SPT).^40^

**Table 2:** Summary of findings of epidemiological studies done in Ecuador of associations between soil‐transmitted helminth (STH) parasites and allergic diseases

Study	Design/population	Sample	Exposures/intervention	Outcomes	Findings
Cooper et al, 2003^27^	Cross‐sectional, Pichincha & Esmeraldas	Schoolchildren, 5‐18 y, n = 4433	Prev: any (63.4%); AL (49.7%); TT (43.8%); Hk (2.3%)	Wheeze 2%, rhinitis 4%; eczema 4%	No significant associations with allergic symptoms
Cooper et al, 2006^29^	Cluster‐randomized trial; bi‐monthly albendazole vs no treatment; Pichincha	Schoolchildren, 7‐17 y, n = 2373 in 68 schools	Bi‐monthly albendazole vs no treatment over 12 mo. Prev: any 72%; AL 56%; Tt 56%; Hk 15%	Wheeze 3%, rhinitis 3%, eczema 4%	No effect of treatment wheeze, rhinitis and eczema after 12 mo
Moncayo et al, 2010^41^	Cross‐sectional, Esmeraldas	Rural schoolchildren, 6‐16 y, n = 3960	Prev: any 75%, Al 53%, Tt 57%, Hk 9%	Wheeze 11%, rhinitis 6%, eczema 5%	No inverse associations with wheeze, rhinitis and eczema. High parasite burden with Tt inversely associated with atopic wheeze (high Tt vs negative, OR 0.24)
Endara et al, 2010^34^	Cross‐sectional, Esmeraldas	Rural schoolchildren, 6‐16 y, n = 3901	Comparison of communities receiving ivermectin MDA for 15‐17 y vs non‐MDA communities. Prev: any 63 vs 86%, Al 49 vs 57%, Tt 31 vs 82%, Hk 15 vs 4%	MDA vs non‐MDA: wheeze 10 vs 11%; rhinitis 6 vs 7%; eczema 7 vs 3%	No significant associations with allergic symptoms. Long‐term MDA associated with higher prevalence of eczema (OR 2.24) but not wheeze or rhinitis. No effect of MDA on association between SPT and allergic symptoms
Moncayo et al, 2013^55^	Case‐control, Esmeraldas	Rural schoolchildren, 7‐19 y, n = 376	Prev: any 72%; Al 47%; Tt 58%; Hk 8%. Anti‐Ascaris IgE 65%	Cases wheeze vs control non‐wheeze	Active STH not associated with wheeze although inverse association between Tt and atopic wheeze (OR 0.47). Anti‐*Ascaris* IgE associated with atopic (OR 6.98) and non‐atopic (OR 2.67) wheeze
Cooper et al, 2014^30^	Cross‐sectional, Esmeraldas	Rural and urban schoolchildren, 5‐16 y, n = 6821	Rural prev: any 69%, Al 42%, TT 54%, Hk 6%. Urban: any 43%, Al 20%, Tt 35%, Hk 5%	Rural: Wheeze 10%, rhinitis 6%, eczema 5% Urban: Wheeze 9%, rhinitis 8%, eczema 6%	Overall no associations with wheeze, rhinitis and eczema. Wheeze associated with Tt (urban OR 1.40 vs rural OR 0.95, *P* inter = 0.04); rhinitis associated with Al (urban OR 0.62 vs rural OR 1.09, *P *= 0.03; Eczema associated with Hk (urban OR 0.50 vs rural OR 1.76, *P *= 0.01)
Endara et al, 2015^53^	Case‐control, Esmeraldas	Rural and urban schoolchildren, 7‐19 y, n = 600	Rural prev: any 73%; Al 47%; Tt 58%. Urban prev: 41%, Al 17%, Tt 32%	Cases wheeze vs control non‐wheeze	No associations with active infections. Presence anti‐*Ascaris* IgE associated with wheeze (urban OR 3.33, rural OR 2.76). PAF% for anti‐*Ascaris* IgE: rural 50% vs urban 35%. Associations between ant‐hdm IgE and wheeze attenuated by geohelminth infection markers
Ardura et al, 2015^54^	Case‐control, Esmeraldas	Urban, children, 5‐15 y, n = 179	Prev: any 4%, Al 2%, Tt 2% Anti‐Ascaris IgE 49%	Acute asthma cases vs non‐asthma controls	Association between asthma and anti‐*Ascaris* IgE (OR 2.24). PAF% for anti‐*Ascaris* IgE 31%
Cooper et al, 2016^26^	Prospective, Esmeraldas	Rural birth cohort to 3 y, n = 2069 follow‐up	Maternal prev: any 46%, Al 28%, Tt 29%, Hk 6%	Any during first 3 y: wheeze, 26%, eczema 18%	No effect of maternal STH on childhood wheeze and eczema to 3 y
Cooper et al, 2018^40^	Prospective, Esmeraldas	Rural birth cohort to 5 y, n = 2090 (of 2404 recruited)	Maternal prev: any 46%, Al 27%, Tt 28%, Hk 6% Childhood prev. to 3 y: any 34%, Al 26%, Tt 17%, Hk 1%	Wheeze 13%, asthma 6%	Maternal STH associated with increased wheeze (OR 1.41). Childhood STH associated with reduced wheeze (OR 0.70) and asthma (OR 0.60). Effects greatest with later age of infection in children and seen only in non‐atopics. Effects not associated with specific parasites or infection intensities

anti‐*Ascaris* IgE, presence of specific IgE to *Ascaris* (≥0.7 kU/L); y, years; MDA, mass drug administration or given at community level; Al, *A. lumbricoides*; Tt, *T. trichiura*, Hk, hookworm; prev., prevalence; hdm, house dust mite; OR, Odds Ratio; P inter, P value for interaction.

Periodic anthelmintic treatments with albendazole over 12 months did not affect the prevalence of allergic symptoms in schoolchildren in Pichincha Province.^29^ These findings are consistent with previous randomized intervention studies done in schoolchildren.^32^, ^33^ Analysis of allergic symptoms among schoolchildren living in communities that had received annual or twice‐annual anthelmintic treatments over the previous 15‐17 years was not associated with a significant difference in wheeze or rhinitis symptoms, but there was an increased risk of eczema symptoms among children living in treated communities.^34^

To investigate if early exposures to STH parasites might affect the development of allergic diseases, we evaluated the ECUAVIDA cohort.^39^ Analyses of the cohort to 3 years did not show an effect of maternal STH infections on wheeze or eczema although in sub‐group analyses maternal ascariasis was associated with an increased risk of eczema in children.^26^ However, by 5 years of age there was an increased risk of wheeze associated with maternal STH infections while childhood STH, particularly those acquired during the third year of life, were associated with a reduced risk of wheeze and asthma, an effect that was only seen among non‐atopic children.^40^ These observed effects were not associated with specific STH parasites or were affected by parasite burden although burdens were low in young children.^40^

How might these contradictory findings be explained? We have shown previously in the same cohort that maternal ascariasis is associated with increased immune responsiveness to *Ascaris* antigens in newborns.^38^ *A. lumbricoides* infections have been shown to increase the risk of wheeze/asthma in older children and adults^7^, ^42^, ^43^, ^44^ and this effect may be “transmitted” from mother to child through in utero sensitization to *Ascaris* antigens. Contrasting effects of STH on wheeze/asthma in younger vs older subjects may depend on history of STH exposures and type of inflammatory lung response. Earlier infections may modulate anti‐parasite inflammatory responses in highly endemic populations^37^ allowing protective effects against wheeze/asthma to appear. Protective immunity to helminth parasites is age‐dependent and non‐sterile in endemic populations.^45^ With continued exposures and immune maturation (eg, around school age), more effective anti‐parasite^45^, ^46^ but heightened inflammatory responses may emerge. Such responses when “transmitted” by infected mothers to their infants may be down‐regulated when early STH exposures are sufficient. Suppression of the anti‐parasite response in early childhood is unlikely to occur in populations with a low prevalence and older age of first infection.

Epidemiological studies generally have detected STH parasites by microscopic examination of single stool samples, an approach that has poor sensitivity among individuals with light infections. An alternative is to measure the presence of parasite‐specific antibodies that indicate past STH exposures, may also indicate current infections, and may be a particularly useful infection marker in populations that have received recent anthelmintic treatments. It has been suggested that measurement of anti‐*Ascaris* IgE is a useful marker for exposure to STH including *A. lumbricoides* in areas of low prevalence^47^ where many more people tend to have this marker compared to those with detectable intestinal infections. However, the presence of anti‐*Ascaris* IgE indicates also allergic sensitization to *Ascaris* and, hence, is probably a measure for atopy or the capacity to respond to parasite allergens with an IgE response. Numerous studies have measured the associations between anti‐*Ascaris* IgE and asthma symptoms, generally showing strong positive associations with wheeze, asthma and bronchial hyper‐reactivity.^48^, ^49^, ^50^, ^51^, ^52^ In cross‐sectional studies in Ecuador, we have observed strong associations between anti‐*Ascaris* IgE and wheeze symptoms among schoolchildren living in rural and urban areas of Esmeraldas Province.^53^, ^54^ Among rural schoolchildren, the presence of anti‐*Ascaris* IgE explained approximately 50% of wheeze symptoms and 30% of wheeze^53^ and asthma^54^ in the City of Esmeraldas. In the case of the latter, only 4% had positive stool samples for STH while 49% had anti‐*Ascaris* IgE.^54^ The presence of anti‐*Ascaris* IgE was associated with IgE sensitization to aeroallergens,^55^ an effect that might be mediated by a higher degree of atopy among wheezy/asthmatic children exposed to ascariasis^49^ or by a direct effect on airways reactivity caused by inflammation consequent to the host response to the presence of *Ascaris* larvae migrating through the lungs.^48^ Alternatively, the association between anti‐*Ascaris* IgE and wheeze could be explained by cross‐reactivity between IgE epitopes of parasites and aeroallergens.^56^ Mite atopy appeared to explain a greater proportion of wheeze in urban compared to rural schoolchildren^53^ and in a population of asthmatics with a low prevalence of STH, the association between anti‐*Ascaris* IgE and acute asthma disappeared after controlling for mite IgE.^54^ This is in contrast to a previous rural study that showed that controlling for mite IgE did not affect the association between anti‐*Ascaris* IgE and wheeze.^55^ Rural wheeze, much of which was explained by anti‐*Ascaris* IgE (and not mite IgE), was a mild self‐limiting illness not requiring maintenance therapy while that in the urban population was more severe, required maintenance therapy more frequently, and was more strongly associated with mite atopy. These data may indicate, therefore, that endemic exposures to ascariasis attenuate asthma severity and that urban populations with a lower prevalence of STH parasites and stronger mite atopy, may suffer more severe disease.

**Table 3:** Summary of findings of immunological studies of effects of soil‐transmitted helminths (STH) on the human immune response and allergic inflammatory responses

Study	Design/population	Sample	Exposures/intervention	Outcomes	Findings
Cooper et al, 2000^60^	Case‐control, Manabi	Teens & adults, 13‐66 y, rural, (n = 113)	*Ascaris*‐infected cases vs uninfected “non‐endemic” controls. STH cases: any 100%, AL 100%, Tt 71%	PBMC cytokine responses by ELISA & ELISPOT to *Ascaris* adult and larval antigens	Infected had greater lymphocyte proliferation, frequencies of PBMCs expressing IL‐4 and IL‐5 and IL‐5 protein production indicative of a highly polarized Th2 response to parasite antigen. No differences for IFN‐γ and IL‐10
Cooper et al, 2004^61^	Cross‐sectional, Pichincha	Schoolchildren, 5‐17 y, rural, (n = 132)	Sample stratified into 4 groups by SPT status and *Ascaris* infection. Population of high prevalence of Al and Tt (ie, >50%)	PBMC cytokine responses by ELISPOT and PBL histamine release to *Ascaris* adult and larval antigens	Elevated histamine release and IL‐4 and IL‐5 expression to larval antigens in SPT+ children, particularly among uninfected
Cooper et al, 2008^66^	Case‐control, Pichincha	Schoolchildren, 7‐13 y, rural, (n = 80)	SPT+ cases vs SPT‐ controls. STH cases: any 43%, AL 23%, Tt 33%, Hk 3%; controls any 30%, AL 15%, Tt 25%, Hk 3%	PBL cultures with parasite antigen or aeroallergen for IL‐10 protein and frequencies of IL‐10+ T cells	No association between SPT+ and Ascaris‐induced IL‐10 and frequencies of IL‐10+ T cells. Immune parameters did not affect association between of hdm‐specific IgE and SPT
Cooper et al, 2008^62^	Cross‐sectional analysis nested within prospective, Pichincha	Schoolchildren, mean age 9 y, rural, (n = 214)	Either treated with albendazole every 2 mo for 12 mo or no treatment. Baseline STH: any 75%, Al 57%, Tt 57%, Hk 7%	Whole blood collected at 12 mo. PBL cultures with parasite antigen or aeroallergen for histamine release (HR) and cytokines	Treatment associated with greater IL‐5 and IL‐13 protein to parasite antigens and SEB in treated children and reduction in IL‐10 protein to *Ascaris*. No effect on HR and aeroallergen‐specific responses
Guadalupe et al, 2009^38^	Case‐control, Esmeraldas	Mothers and newborns, rural, (n = 28)	Newborn of infected mother cases vs newborn of uninfected mother controls. STH infected mothers: any 100%, AL 100%, Tt 79%, Hk 29%	Cord blood frequencies of CD4+ T cells positive for IFN‐γ or IL‐4 from PBL cultures stimulated with *Ascaris* antigens	Higher frequencies of IL‐4+ and IFN‐g+ CD4+ T cells in cord blood of newborns from infected mothers
Teran et al, 2011^81^	Cross‐sectional, Esmeraldas	Children, 6 mo‐5 y), (n = 240)	STH, urban at 4‐5 y: any 43%, Al 23%, Tt 37%; rural, any 54%, Al21%, Tt 54%	PBL cytokine responses to SEB and TLR agonists. Frequencies of Tregs	Area of residence but not STH infections in children at 6‐9 mo had significant effects on outcomes. Age‐dependent down‐regulation of IL‐10
Reina Ortiz et al, 2011^74^	Cross‐sectional, Esmeraldas	Schoolchildren, 7‐12 y, rural (n = 60)	Children classified as uninfected, light‐ and chronically‐infected. STH chronic: Any 100%, Al 100%, Tt 100%	PBMCs cultured in medium alone and gene expression on unstimulated PBLs	Chronic STH associated with greater production of cytokine protein incl. IL‐5 and IL‐10. Evidence of differential gene regulation: chronic infections greater expression of homeostatic genes (eg, IDO)
Larson et al, 2012^68^	Prospective, Esmeraldas	Schoolchildren, 8‐12 y, rural (n = 22)	STH prev.: any 100%, Al 100%, Tt 100%	Whole blood histamine release to anti‐IgE before vs 14 d after treatment	Basophil activation by anti‐IgE increased post‐treatment
Cooper et al, 2015^77^	Cross‐sectional, Esmeraldas	Schoolchildren, 6‐19 y, urban and rural n = 440	STH prev.: urban, any 21%, Al 10%, Tt 17%; Rural, any 74%, Al50%, Tt 62%	PBL cytokine responses to medium alone (homeostasis), *Ascaris*, hdm, and SEB	Urban children more likely to produce IL‐10 to multiple stimuli. SEB‐induced IL‐10 partly explained by STH in urban but not rural schoolchildren
Figueiredo et al, 2016^78^	Cross‐sectional, Esmeraldas	Schoolchildren, 6‐19 y, urban and rural n = 310	STH prev. (any 51%) and other poor hygiene exposures	Innate and adaptive cytokines by PBLs stimulated with media alone and SEB (maximal stimulation)	Cytokine response phenotypes defined by latent class analysis. Phenotypes: innate—low vs high; adaptive—low vs modified Th2. STH associated with regulated phenotypes

PBMC, peripheral blood mononuclear cells; PBL, peripheral blood leukocytes; SEB, *Staphylococcus* enterotoxin B; SPT+/− allergen skin prick test positive/negative; Tregs, regulatory T cells; anti‐*Ascaris* IgE, presence of specific IgE to *Ascaris* (≥0.7 kU/L); y, years; mo, months; IDO, indoleamine oxidase; MDA, mass drug administration or given at community level; Al, *A. lumbricoides*; Tt, *T. trichiura*, Hk, hookworm; prev., prevalence; hdm, house dust mite; OR, Odds Ratio.

Soil‐transmitted helminths parasites might affect the expression and severity of wheeze/asthma through the attenuation of the association with atopy. There is evidence from other regions, that STH parasites may weaken the association between atopy and allergic symptoms.^57^, ^58^ In case‐control studies done among rural and urban schoolchildren in Esmeraldas Province, we showed that mite atopy (measured by IgE) was more strongly associated with recent wheeze in urban compared to rural children and that the association between atopy and wheeze was attenuated by markers of STH infection.^53^

Overall, data from a series of cross‐sectional studies conducted in Ecuador have not shown consistent effects of STH on allergic diseases. However, 3 observations from these studies are of particular interest: (a) the role of anti‐*Ascaris* IgE as an important risk factor for a mild wheezing illness in rural populations where STH are endemic; (b) the role of STH infections in attenuating the associations between atopy and wheeze; and (c) the potential role of maternal and childhood STH infections in modulating risk of wheezing illness by school age.

## 7 IMMUNOLOGICAL MECHANISMS MEDIATING STH EFFECT ON HOST INFLAMMATORY RESPONSE

Our observations to date of effects of STH parasites on atopy and allergic diseases in Ecuador indicate a complex relationship. The inverse association between STH parasites and SPT has been interpreted to suggest an active suppressive mechanism but our observations indicate, that if real, this relationship may take many years of living in a parasite‐free or less endemic environment to be reversed.^34^ Observations from Europe indicate that emergence of atopy, once a protective farming‐related exposure has been removed, may occur over a period of years and is largely independent of age.^59^

Findings of studies investigating immunological mechanisms are summarized in Table 3. Although STH parasites induce strong Th2 response in humans,^60^ the intensity of the response seems to be modulated by concurrent STH infections^61^ and increases following effective treatment.^62^ Th2‐mediated inflammatory responses are required to kill and expel parasites and the ability of the parasites to modulate the severity of these responses has clear implications for parasite survival.^25^ The mechanisms by which STH parasites modulate host inflammatory responses are uncertain. The anti‐inflammatory cytokine, IL‐10, has a powerful immune modulatory role in tissue helminth infections such as filarial and schistosome parasites,^63^ but its role during STH infections is less clear.^25^, ^60^

The suppression of SPT associated with STH parasites may be explained by a reduction in the responsiveness of cells that bear the high‐affinity receptor for IgE (ie, mast cells and basophils) to activation signals including cross‐linking of specific IgE. An effect of this suppression is the marked disassociation between the presence of specific IgE and SPT to specific allergens that has been reported in numerous STH endemic populations including in Ecuador.^55^ Explanations for the disassociation include immunological suppression through immune mediators such as IL‐10^64^ and the presence of IgE of low biological activity directed against cross‐reactive protein and carbohydrate structures in STH parasites.^65^ We have explored potential mechanisms by which STH infections might suppress SPT and were unable to identify a role for parasite‐induced IL‐10^66^ in suppressing SPT or the disassociation between specific IgE and skin test reactivity to specific allergens,^66^ reported previously for schistosomiasis.^64^ It has been suggested that high levels of polyclonal IgE may modulate immediate hypersensitivity reactions by “saturation” of high‐affinity IgE receptors (IgE‐FεRI) on mast cells—we have observed a reduced prevalence of SPT among children with high levels of polyclonal IgE (ie, >3561 IU/mL). However, we observed a similar inverse association with SPT for the presence of anti‐*Ascaris* IgG4.^26^ Both total IgE and anti‐*Ascaris* IgG4 are likely to be associated with chronic STH infections and may be markers for other immunological factors associated with chronic infections that mediate protection against SPT. Further, it has been shown that polyclonal IgE levels during helminth infections are rarely high enough to inhibit allergen‐specific IgE‐FεRI binding and to suppress allergen‐induced degranulation of mast cells and basophils.^67^ We were able to show that basophil reactivity to IgE‐dependent (anti‐IgE) and IgE‐independent (ionomycin) stimuli increased rapidly following treatment of STH parasites.^68^ However, we were unable to detect specific effects of long‐term anthelmintic treatment on basophil or Th2 responses to aeroallergens.^62^ A study in Indonesia provided evidence that STH parasites may suppress cellular hyporesponsiveness through increased expression of inhibitory CTLA‐4 on CD4+ T cells^69^ although it is not clear if this mechanism could affect immediate hypersensitivity responses.

Helminths parasites are not alone in being associated inversely with atopy.^70^ Infections with several childhood infections have been associated with a similar inverse association.^70^, ^71^ These include a variety of bacterial, viral and parasitic infections^70^ as well as a variety of exposures associated with poverty and poor hygiene such as household hygiene,^72^ exposure to farm animals^59^, ^73^ and household overcrowding.^28^ Thus, the helminth effect on SPT might not be mediated by STH but by exposures with which they are associated or the effect might be one for which STH are sufficient but not necessary. As mentioned above, it appears unlikely that STH‐specific IL‐10 has a key role in modulatory effects of STH on non‐parasite inflammatory responses such as that measured by SPT.

Soil‐transmitted helminths infections have been associated with a stronger regulation of the immune response that can be measured in vitro either at homeostatis (ie, that measured in unstimulated cultures of peripheral blood leukocytes [PBLs]) or upon maximal stimulation (ie, after mitogen stimulation of PBLs). For example, STH infections have been associated with greater levels of homeostatic IL‐10^74^, ^75^, ^76^ and expression of homeostatic genes^74^ that may contribute to a more robust regulation of inflammatory responses in a non‐specific manner in the context of a wide array of poor hygiene exposures with similar effects.^75^, ^77^, ^78^, ^79^, ^80^ The immune systems of children living in poor environments in LMICs are likely to be exposed to a wide variety of infectious disease insults. For example, we have observed that young children up to 5 years of age in rural and marginal urban areas of Ecuador show a rapid development of the memory T cell response and down‐regulation of innate inflammatory responses and anti‐inflammatory responses measured by IL‐10.^81^ Studies including those using unbiased clustering methods to define phenotypes of host immune response indicate that STH infections are associated with more regulated immune phenotypes^74^, ^78^ and that such immune phenotypes are associated with a lower risk of atopy.^72^ These phenotypes do not appear to be explained solely by the presence of STH parasites but by multiple poor hygiene and poverty‐related exposures within a child's living environment.^72^, ^75^

## 8 CONCLUSION

There is an unresolved debate on whether STH parasites protect against allergy and allergic diseases including asthma, potentially of great importance in populations where improvements in hygiene and widespread use of anthelmintic drugs are expected to reduce the prevalence of STH prevalence dramatically over the coming years. We have reviewed a series of epidemiological and immunological studies done in Ecuador of the effects of endemic STH parasites on the development of allergic diseases and the allergic inflammatory response. Data from cross‐sectional studies of schoolchildren in regions that are highly endemic for the STH parasites, *A. lumbricoides* and *T. trichiura,* have consistently shown inverse associations between the presence of these STH parasites and a reduced risk of allergen skin prick test (SPT) reactivity. However, a prospective study from birth and a randomized intervention study in which schoolchildren were dewormed were unable to show a measurable effect of STH parasite infections on the development or presence of SPT, respectively. Similarly, the presence of STH infections did not appear to affect the risk of allergic symptoms although they did attenuate the association of allergic symptoms with atopy, and allergic sensitization to *Ascaris* appeared to explain a significant proportion of mild wheezing illness observed in rural populations. Findings from a birth cohort did, however, indicate that the acquisition of STH infections in early childhood might reduce the risk of wheezing illness in later childhood, but it remains uncertain if such protective effects persist beyond early childhood. Analyses of the effects of STH infections on host immune and inflammatory responses show that these parasites do modulate the host anti‐parasite immune response and are likely contribute to tighter regulation of the inflammatory response but in the context of a wide variety of other environmental exposures. Overall, these data indicate that potentially protective effects of STH parasites against wheeze may be present in early childhood in endemic regions of rural Ecuador, but such effects may not persist beyond school age at which time the development of allergic sensitization to *Ascaris* may contribute to a mild but self‐limiting asthmatic illness.
